# Ayurvedic management of Non-Sjögren's Dry Eye (*Shushkakshipaka*) as a preparation for cataract surgery—a case report

**DOI:** 10.3389/fmed.2026.1909897

**Published:** 2026-09-14

**Authors:** Sushma Naranappa Salethoor, Anil Radhakrishnan, Shyamasundaran Kulangara, Ramya Shruthi Janardhanan, Rammanohar Puthiyedath, Reshma NS

**Affiliations:** 1 Department of Shalakya Tantra, Amrita School of Ayurveda, Amrita Centre for Advanced Research in Ayurveda, Amrita Vishwa Vidyapeetham, Kollam, India; 2 Department of Ophthalmology, Amrita Institute of Medical Sciences, Amrita Vishwa Vidyapeetham, Kochi, India; 3 Department of Samhita, Siddhanta and Sanskrit, Amrita School of Ayurveda, Amrita Centre for Advanced Research in Ayurveda, Amrita Vishwa Vidyapeetham, Kollam, India; 4 Department of Shalakya Tantra, Amrita School of Ayurveda, Amrita Vishwa Vidyapeetham, Kollam, India; 5 Amrita Centre for Advanced Research in Ayurveda, Amrita School of Ayurveda, Amrita Vishwa Vidyapeetham, Kollam, India; 6 Department of Psychiatry, Kasturba Medical College Mangalore, Manipal Academy of Higher Education, Manipal, India

**Keywords:** aqueous deficiency, Ayurveda, case report, integrative approach, Non-Sjögren's Dry Eye, ocular surface disease (OSD), *Shushkakshipaka*

## Abstract

This case report describes *Shushkakshipaka* (Non-Sjögren's Aqueous Deficient Dry Eye – Non-Sjögren's ADDE) in a patient unfit for cataract surgery due to compromised ocular surface integrity. A 73-year-old woman presented with ocular irritation, foreign body sensation, and blurred distant vision for three months. She had a six-year history of type II diabetes mellitus and hypertension, controlled. Evaluation revealed Grade 2 Nuclear Sclerotic cataract (NS) and Posterior Subcapsular cataract (PSC) (Left > Right) with Punctate Keratopathy, making immediate bilateral cataract surgery unsuitable. Schirmer's test I showed right eye <1 mm and left eye <1 mm, and tear break-up time (TBUT) was right eye (4 s) and left eye (6 s). She was diagnosed with moderate-to-severe non-Sjögren's ADDE and cataract. During ophthalmic evaluation, the patient was diagnosed with punctate keratopathy and dry eye symptoms. She was prescribed artificial tears and omega-3 fatty acid supplements. Cataract surgery was deferred, as the patient was unfit for surgery. Owing to inconsistent treatment adherence, conditions showed minimal improvement at the time of presentation to the Ayurveda hospital. She was diagnosed as *Vata-Pitta Pradhana Shushkakshipaka* (predominance of *Vata* and *Pitta Doshas*; in Ayurveda, *Vata, Pitta* and *Kapha* are collectively called *Tridosha,* the three regulatory functional factors of the body). Treatment had three phases. The first focused on systemic intervention, with *Deepana* (metabolism-enhancing) and *Pachana* (digesting), followed by *Mrdu Shodhana* (mild therapeutic bio-cleansing) and *Marsha Nasya* (high-dose errhine therapy). Following systemic cleansing and attainment of *Niramavastha* (a state free from accumulated metabolites achieved by either therapeutic measures or the effect of time), the second phase focused on local ocular management through *Netra Kriyakalpa* (localised ocular therapeutic procedures). The third involved follow-up and maintenance therapy for long-term ocular stability. Oral Ayurvedic polyherbal formulations, dietary modifications, and lifestyle changes were administered throughout the two-month Ayurveda treatment period. After two months, parameters improved, with Schirmer's values of right eye 5 mm and left eye 7 mm and TBUT of right eye 8 s and left eye 14 s, making her fit for cataract surgery. Within the Ayurvedic framework, these changes may be viewed as consistent with improved *Agni* (digestive/metabolic factors) and supportive of subsequent *Kriyakalpa*; links with ocular surface integrity or the gut-eye connection remain hypothetical. This case warrants further investigation in selected cataract surgery candidates with severe, non-responsive dry eye.

## 1 Introduction

The Lancet Commission for Global Surgery estimates that about 11% of the global burden of diseases requires surgical management. Among them, cataract surgery is the most commonly performed operation, especially in the elderly population (1). Exacerbation of pre-existing dry eye symptoms is common in patients who undergo cataract surgery. Proper evaluation of dry eye to determine the severity and aggressive management are undertaken before surgery to deter complications like corneal ulcers. Yet, in severe dry eye, which is one of the ocular surface diseases, patients fail to respond to conventional lubricants due to multiple factors, and such patients are unable to undergo cataract surgery (2). Optimisation of the ocular surface before cataract surgery remains a clinical challenge, particularly when refractory cases respond inadequately to conventional lubricant-based care. Such scenarios highlight the need to document and evaluate individualised Ayurvedic management strategies, particularly in cases where systemic symptoms are addressed alongside ocular treatment. Dry eye manifests as aqueous-deficient type, caused by inadequate tear production due to conditions like Sjögren's syndrome, lacrimal gland dysfunction, obstruction, or systemic drugs. Evaporative dry eye, on the other hand, results from increased tear film evaporation, often due to meibomian gland dysfunction, despite normal tear production (3). Sjögren's disease (SjD; formerly Sjögren's syndrome) is an autoimmune disorder affecting the salivary and lacrimal glands, leading to dry mouth and dry eye symptoms (4). In contrast, non-Sjögren's (sicca syndrome) has a heterogeneous, non-autoimmune aetiology (5). This non-Sjögren's syndrome-related dry eye is an aqueous-deficient subtype characterised by decreased tear production and a Schirmer's test score of less than 5 mm, without the presence of Sjögren's syndrome (6).

This case report presents a case resulting from lacrimal dysfunction, the most common cause of which is age-related lacrimal gland deficiency. Emerging biomedical evidence suggests an association between inflammation, gut dysbiosis, and several disease conditions, and non-Sjögren's aqueous deficiency dry eye is no exception (7). There is also a prominent school of thought advocating the utilisation of omega-3 fatty acids in dry eye treatment, purportedly acting by modulating the gut microbiome (8). Dry eyes cause reduced tear film volume, hyperosmolarity and inflammation in the ocular surface and are among the most common reasons for a visit to an ophthalmology clinic (9). Around the world, it is estimated that between 5% and 34% of people have some form of dry eye, and prevalence significantly increases with age (10). Symptoms improve with treatment, but the condition tends to be long-standing. It is a chronic and potentially sight-threatening condition that occurs due to the dysfunction of the lacrimal glands (11). Emerging evidence suggests that Western dietary patterns, characterised by high intake of refined carbohydrates, saturated fats, and meat, may adversely influence gut microbiome diversity, gut dysbiosis, and inflammatory status (12).

An Ayurvedic approach addressing both digestive and metabolic factors and ocular manifestations may offer a potential management strategy for eye diseases and warrants further investigation. According to the classical Ayurveda text *Ashtanga Hridayam,* the *Samprapti* (pathogenesis) of *Netra Roga* (eye disease) is the consequence of deranged *Dosha* (regulatory functional factors of the body) from *Koshta* (viscera) reaching various parts of *Akshi* (eye) through *Urdhwagata Siras* (any tubular vessels of the body towards the head) (13). In *Charaka Samhita*, while discussing the condition of *Udavarta* [upward movement of *Vayu* (air element) and retention of stool and urine], it is mentioned that *Netra Roga* can manifest as one of its *Upadrava* (complications) (14), thereby indicating a potential gut-eye relationship. The *Pitta Dosha* (the *Dosha* responsible for regulating body temperature and metabolic activities) and *Agni* (digestive/metabolic factors) play an influential role in *Netra Roga* due to *Guna Samanya Sambandha* (similarity at the level of the qualities). The eyes are primarily governed by *Alochaka Pitta* (one of the five subtypes of *Pitta* responsible for vision and perception), while *Pachaka Pitta* (located between the stomach and large intestine) regulates the function of all other *Pitta* subtypes (15). Also, deranged digestive/metabolic regulation is the root cause of various diseases, especially of the gut. Thus, correcting the underlying factors of the pathogenesis*,* which takes place in the gut, and then/simultaneously treating the eye with local *Kriyakalpa* after this renders better relief in ocular conditions as well. This case report highlights an approach focusing on both the gut and the eye while managing non-Sjögren's ADDE (Aqueous Deficient Dry Eye), emphasising the relevance of addressing gut imbalance along with ocular treatments rather than only treating the eye. As a result of this, the patient was able to undergo cataract surgery after Ayurvedic intervention.

## 2 Patient information

### 2.1 De-identified patient data

A 73-year-old female, native of and living in *Kerala*, presented at our hospital in *Kollam, Kerala, India,* seeking Ayurvedic treatment for her dry eye complaints.

### 2.2 Patient concerns and symptoms

The patient presented with symptoms of ocular irritation, foreign body sensation, burning sensation and blurring of distant vision in both eyes and approached the Department of Ophthalmology of a biomedical hospital, where she was found to be suffering from bilateral grade 2 nuclear sclerotic cataract (NS) along with posterior subcapsular cataract (PSC), which had progressed more in the left eye than the right, and was advised to undergo cataract surgery. During the pre-operative examination, it was found that the patient was suffering from severe dry eye, with corneal staining revealing punctate keratopathies with mucus debris. Hence, the scheduled surgery was cancelled due to a poor surgical prognosis in ocular surface disease.

She sought Ayurveda treatment because of the persistence of ocular irritation, foreign body sensation, burning sensation and blurring of distant vision in both eyes associated with constipation and tiredness over the past three months despite the biomedical treatment. She also wanted to reduce her dependency on eye lubricants and reported that her cataract surgery was postponed due to the persistence of symptoms related to dry eye and reduced ocular surface index.

### 2.3 Medical, family, psychosocial history

The patient is a known case of type II diabetes mellitus and grade 1 diastolic dysfunction and has been hypertensive for six years. She was under Tab. Metformin 500 mg for diabetes mellitus and Tab. Losartan 50 mg for hypertension for the past six years. She occasionally used Tab. Neurobion for vitamin B-complex supplementation. The patient had a hysterectomy done at the age of 37.

### 2.4 Past interventions and outcomes

She was prescribed Optive® [Carboxymethylcellulose (CMC) sodium 0.5%] eye drops: 1 drop in each eye 4–6 times daily for 3 months and Omega-3 fatty acid: 1,000 mg/day oral supplements and was advised to revisit for an evaluation after the completed course. After three months, the patient's condition did not adequately improve due to inconsistent adherence to the advised treatment with the drops and oral supplements, and she approached Ayurvedic management of her condition.

## 3 Clinical findings

### 3.1 During the first consultation at the biomedical hospital (on 25th July, 2022)

The patient's visual acuity was measured as 6/60 in both eyes, with pinhole correction to 6/6. The prescription for glasses was +2.75 Ds for the right eye and +3.00 Ds for the left eye, both corrected to 6/6, with an additional +2.75 Ds for near vision in both eyes (N8). Non-contact tonometry recorded intraocular pressures of 19 mmHg in the right eye and 16 mmHg in the left eye. Keratometry readings were 43.25/43.50 at 80/170 with a cylindrical correction of −0.25*80 for the right eye and 43.50/44.25 at 85/175 with a cylindrical correction of −0.75*85 for the left eye. Lens examination indicated grade 2 NS with an immature senile cataract (IMSC) in both eyes. Meibography revealed tortuous but normal glands. Schirmer's test I showed the values in the right eye 2 mm and the left eye 3 mm. Slit lamp examination with corneal staining revealed punctate keratopathies with mucus debris. The diagnosis was based on these clinical observations, and a confirmatory meibography was conducted at the biomedical hospital to exclude meibomian gland dysfunction.

### 3.2 Clinical findings at ayurveda hospital (on 27th October, 2022)

A slit lamp examination showed a tear film break-up time of 4 s for the right eye and 6 s for the left eye. Schirmer's test I results were <1 mm for the right eye and <1 mm for the left eye. No clinically evident punctate keratopathy was observed on examination at our hospital. Mild congestion was observed in both bulbar conjunctivae, along with concretions in the palpebral conjunctivae of the lower lid of both eyes. Senile arcus was present. Refer to Table 1 for specific details of slit lamp examination.

**Table 1:** Slit lamp examination findings.

O/E	Right Eye	Left Eye
Eyelid	Within normal limits, Normal Meibomian gland orifice.	Within normal limits, Normal Meibomian gland orifice.
Conjunctiva	Mild congestion in bulbar conjunctiva, concretions present in palpebral conjunctiva of the lower lid.	Mild congestion in bulbar conjunctiva concretions present in palpebral conjunctiva of the lower lid.
Sclera	White	White
Cornea	Senile arcus present, lustreless	Senile arcus present, lustreless.
Lens	Grade 2 Nuclear Sclerotic Cataract along with Posterior Subcapsular Cataract	Grade 2 Nuclear Sclerotic Cataract along with Posterior Subcapsular Cataract
Pupil	Small size, sluggish reaction	Small size, sluggish reaction
TBUT	Tear film break-up time of 4 s for the right eye.	Tear film break-up time of 6 s for the left eye.
Schirmer's test 1	Right Eye <1mm	Left Eye <1mm

## 4 Timeline

The patient underwent a three-month course of biomedical intervention, followed by two months of exclusive *Ayurvedic* treatment. Refer to Table 2 and Figure 1 for details on specific dates, complaints, interventions and outcomes.

**Table 2:** Timeline.

Prior treatments			
A 73-year-old female with a known case of Type 2 DM, HTN in the last 6 years with normal meibography, Schirmer's test I in right eye < 1 mm and left eye < 1 mm with bilateral grade 2 Nuclear Sclerosis and Posterior Subcapsular Cataract suffering from irritation, blurring of vision, foreign body sensation and constipation, sought Ayurvedic treatment.			
Date	Patient complaints	Ayurvedic intervention	Biomedical/other Interventions
25/07/2022	During ophthalmic consultation, Bilateral Grade 2 Nuclear Sclerosis with Posterior Subscapular Cataract was diagnosed, recommending cataract surgery. However, ophthalmology workup revealed severe dry eye: right eye 2 mm, left eye 3 mm. Surgery was postponed.	Not administered	She was prescribed omega supplements and artificial tear drops and advised to visit after a month but even after 3 months of using teardrops and the patient didn't get much relief.
**Main treatments**			
27/10/2022	She presented with symptoms of irritation, watering of the eye and foreign body sensation, burning sensation associated with constipation and tiredness. The Schirmer's test I values were Right Eye <1 mm, Left Eye <1mm	*Ayurvedic* internal Medications aiming *Deepana* and *Pachana* were started with *Amritottara Kashayam, Vaishwanara Churnam*	No biomedical interventions.
17/11/2022	Watering of the eye relieved. Schirmer's test I at the end of the treatment – Right: 2 mm, Left: 4 mm.	*Shamana Snehapana, Sarvanga Abhyanga, Bashpa Sveda, Mrdu Virechana, Nasya* followed by *Netra Kriyakalpas, Seka, Anjana and Ashchyotana*	No biomedical interventions.
18/01/2023	Schirmer's test I at the end of the *Tarpana* treatment Right Eye 6 mm, Left Eye 8 mm, TBUT - Right Eye - 3Sec, Left Eye - 14Sec.	Tarpana treatment	No biomedical interventions.
**Follow-up**			
16/02/2023	Patient had better clarity in vision, foreign body sensation reduced, Schirmer's test I – Right: 6 mm; Left: 8 mm, TBUT – Right: 8 Sec, Left: 14 Sec.	*Anu Tailam* for *Pratimarsha Nasya*, Eye drops – *Shatahwadi Ghritam*, *Sreedhareeyam* Eyeplus eye drops	No biomedical interventions.
22/03/2023	Consulted a biomedical ophthalmologist.	*Shatahwadi* eye drops once a week, *Anu Tailam* for *Pratimarsha Nasya* daily, but stopped all Ayurveda interventions for a short while from the time of surgery to the completion of the surgery follow-up.	Posted for the Cataract surgery
22/04/2024	After the cataract surgery, the patient was complaining of symptoms of dry eyes, Schirmer's test I – Right Eye 2 mm, Left Eye 4 mm; TBUT – Right Eye - 5Sec, Left Eye - 5Sec	Started *Ashchyotana* with *Shatahwadi* Eye drops for a week.	No biomedical interventions.
07/02/2025	Schirmer's test I - Right Eye - 6 mm, Left Eye - 8 mm, no discomfort	No Ayurveda medications given	No biomedical interventions.

**Figure 1:**
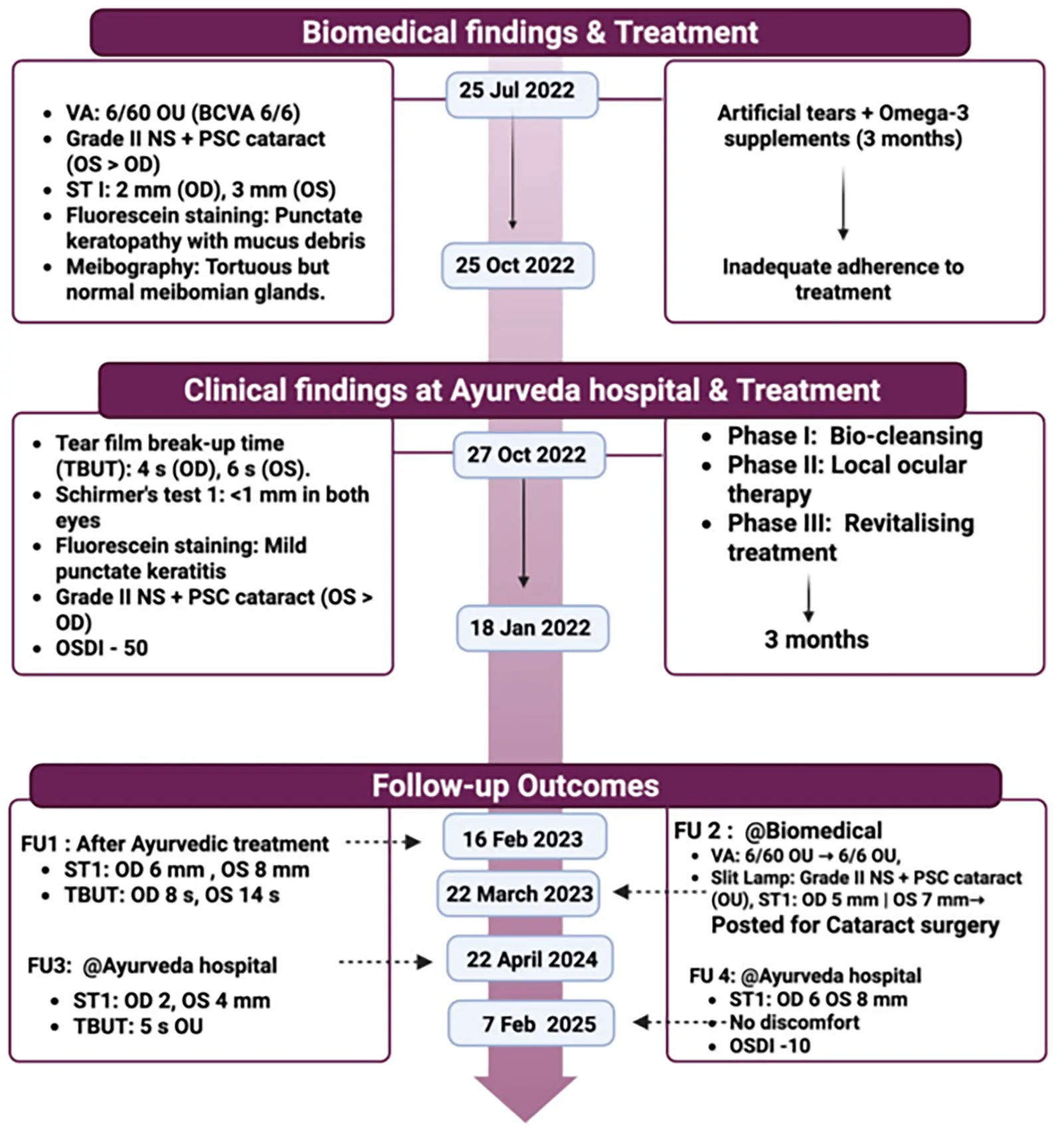
Graphical presentation of the timeline.

## 5 Diagnostic assessment

### 5.1 Diagnostic methods

The biomedical diagnosis was based on clinical observations and comprehensive ocular diagnostics for dry eye. These tests included assessing the patient, evaluating the symptoms and reviewing medical history. A tear film assessment was conducted using Schirmer's test I and TBUT, which are routine tests done to clinically diagnose dry eye. The ocular surface was examined with a slit lamp, and corneal and conjunctival staining with sodium fluorescein highlighted areas of damage or dryness. Meibomian gland evaluation was performed through meibography and manual expression. Functional vision tests were also conducted. Refer to Table 3 and Figure 2 for the timeline of Schirmer's and TBUT tests and their values. The OSDI score before the start of Ayurveda treatment was 50, which falls under the severe dry eye category.

**Table 3:** Timeline of Schirmer's test 1 and TBUT tests.

Sl. No.	Date	Schirmer's test	TBUT	Interventions
1	25/07/2022	Right Eye 2 mm, Left Eye 3 mm	-	During biomedical ophthalmology consultation
2	27/10/2022	Right Eye <1 mm, Left Eye <1mm	Right Eye - 4 Sec, Left Eye - 6 Sec	1^s^day of Ayurveda consultation
3	03/11/2022	Right Eye 1 mm, Left Eye 1 mm	-	End of *Deepana Pachana* treatment
4	25/11/2022	Right Eye 1 mm, Left Eye 1 mm	-	After *Snehapana* and *Mrdu Virechana*
5	12/12/2022	Right Eye 2 mm, Light Eye 4 mm	-	After *Marsha Nasya* and *Sthanika Netra Kriyakalpas (Seka and Ashchyotana)*
6	30/12/2022	Right Eye 5 mm, Left Eye 6 mm	-	Oral medications were given along with *Ashchyotana* and *Pratimarsha Nasya*
7	12/01/2023	Right Eye 5 mm, Left Eye 6 mm	Right Eye - 5Sec, Left Eye - 5Sec	Oral medications were given along with *Ashchyotana* and *Pratimarsha Nasya*
8	16/02/2023	Right Eye 6 mm, Left Eye 8 mm	Right Eye - 8Sec, Left Eye - 14Sec	After one month of follow-up
9	22/03/2023	Right Eye 5 mm, Left Eye 7 mm	-	Posted for the Cataract surgery
10	22/04/2024	Right Eye 2 mm, Left Eye 4 mm	Right Eye - 5Sec, Left Eye - 5Sec	Follow-up after cataract surgery. *Ashchyotana* was done.
11	07/02/2025	Right Eye 6, mm Left Eye 8 mm	-	Latest follow-up.

**Figure 2:**
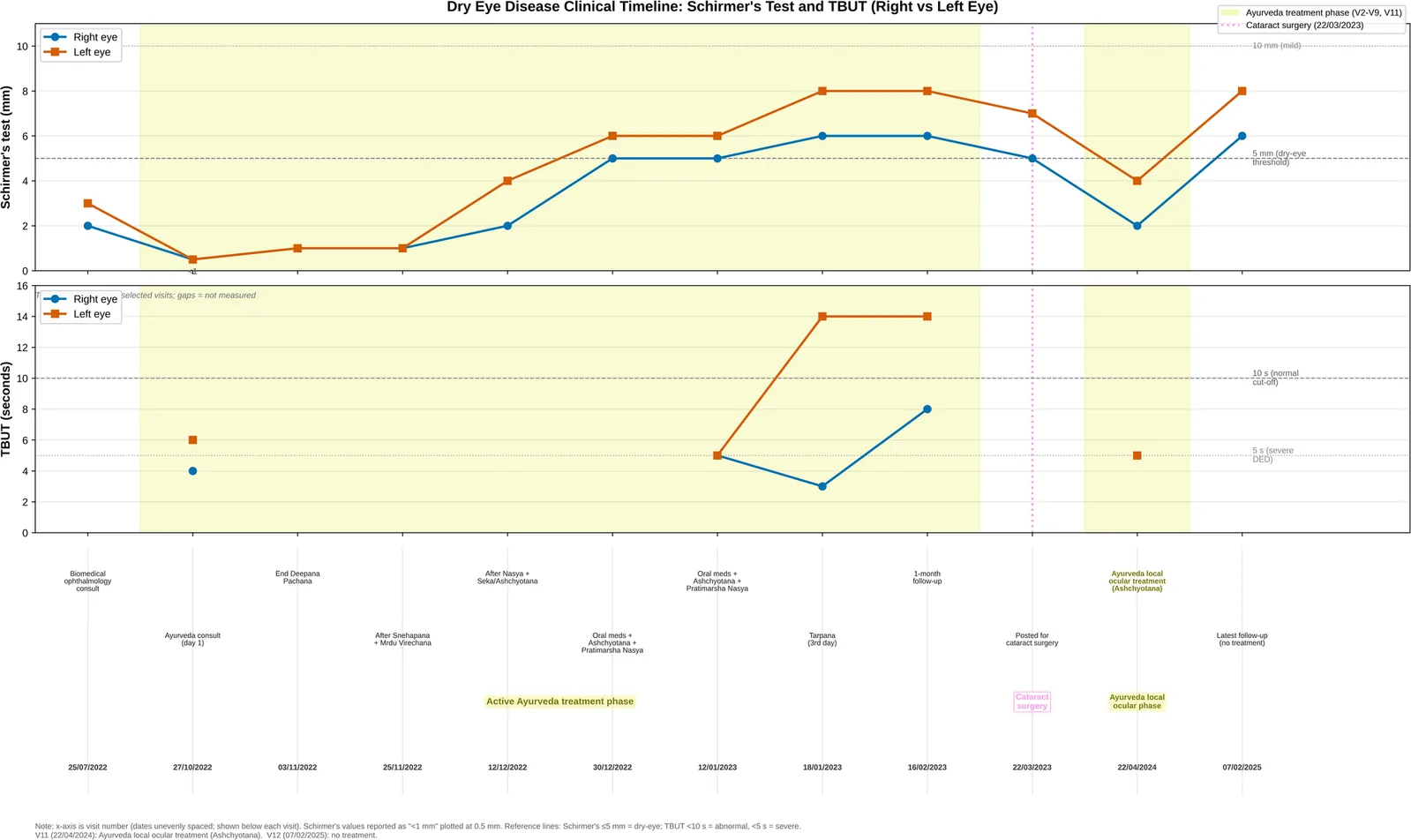
Timeline of Schirmer's Test 1 and TBUT tests.

### 5.2 Diagnostic challenges

There were diagnostic challenges encountered, especially during the documentation for this case report, as only the required and routine tests for clinical evaluation were done and it was not rigorous research-level documentation. However, the biomedical diagnosis was already established by a biomedical ophthalmologist. From the symptoms of the patients, examination of the patient and diagnostic features, the Ayurveda diagnosis of *Vata-Pitta Pradhana Shushkakshipaka* (*Shushkakshipaka* with a predominance of *Vata* and *Pitta Doshas*) was arrived at.

### 5.3 Diagnosis

#### 5.3.1 Biomedical diagnosis

Although tear film instability was evident from the reduced TBUT, there were no clinical findings suggestive of meibomian gland dysfunction or other evaporative mechanisms to support a diagnosis of evaporative or mixed dry eye. Furthermore, in the absence of clinical features of Sjögren's syndrome, the case was classified as non-Sjögren's aqueous-deficient dry eye. Old age and other systemic illnesses which are present in this case also affirm the diagnosis (7). Thus, this clinical presentation and the clinical findings pointed to the case as severe non-Sjögren's aqueous deficiency dry eye (16).

#### 5.3.2 Ayurvedic diagnosis

Initial diagnostic considerations included *Krichronmeelana* (17), a *Vartmagata Roga* (eyelid disorder), based on the presence of symptoms consistent with *Pamshu Purna Netra* (foreign body sensation) and *Krichronmeelana* (difficulty in eyelid opening). However, a critical diagnostic symptom for *Krichronmeelana*, symptomatic and momentary relief upon ocular rubbing, was notably absent in this case. Conversely, *Shushkakshipaka* does not typically present with this feature. The absence of symptomatic relief upon ocular rubbing in our patient led to the exclusion of *Krichronmeelana* as a diagnosis.

Upon thorough evaluation and comparison with the aforementioned conditions, we have identified *Gharsha* (foreign body sensation), *Toda* (pricking pain), *Upadeha* (mucous debris), *Avila Darshana* (blurring of vision), *Ruksha Daruna Vartma* (crusting of lids), and *Sheeteccha* (desire for cold things) as prominent symptoms indicating aggravated *Vata*. Additionally, *Paka* (inflammation) and *Daha* (burning sensation) suggest aggravated *Pitta*. Consequently, the condition is considered *Vata-Pittaja Sushkakshipaka* (18), which is classified as a *Bheshaja Sadya Vyadhi* (classified in Ayurveda as a condition treatable with oral medications and localised treatments) (19).

### 5.4 Prognosis

Owing to the chronicity, ADDE can lead to ocular surface complications like punctate keratopathy, which can potentially threaten vision. Additionally, ageing, female sex, and diabetes form the most common risk factors for ADDE. In this case, the predisposition of risk factors and inadequate adherence might be a contributing factor to reduced treatment outcomes of the standard-of-care intervention. Judicious employment of topical lubricants, anti-inflammatory agents, secretagogues, and autologous serum are generally utilised along with systemic therapy, including immunomodulators, in the treatment of dry eye disease. Long-term treatment for ADDE is imperative to prevent keratopathy and blindness (20, 21).

## 6 Therapeutic intervention

### 6.1 Phases of therapeutic intervention

The therapeutic intervention was planned in three phases (22, 23), the first focusing on correcting the gut, which included the treatments starting from *Deepana* (metabolism-enhancing), *Pachana* (digesting), and *Mrdu* (mild bio-cleansing), both *Urdhwa* and *Adho Shodhana* (bio-cleansing from both upper and lower parts of the body).

The second phase of treatment focused on local ocular management, starting from *Lekhana* (cleansing) to *Brmhana* (nourishment), which was the line of treatment chosen for the management of this condition.

In the third phase, as a follow-up, maintenance and strengthening, *Anulomana* (mild purgation) was done. Specific eye treatments for *Prasadana* (quality-enhancing) and *Rasayana* (revitalising) were also administered for the maintenance and strengthening.

Considering the age, the digestive capacity and the tolerance capacity of the patient to take up Ayurvedic treatment, we intended to do only a *Mrdu Shodhana* and hence fixed a mild dose.

### 6.2 Administration of therapeutic intervention

#### 6.2.1 Internal medications

The medications administered were *Amritottaram Kashayam, Vaishwanara Churnam, Indukanta Ghritam, Gandharvahastadi Eranda Tailam* and *Drakshadi Kashayam*.

Refer to Table 4 for a complete list of internal medications.

**Table 4:** List of internal medications.

Internal medications				
Date	Intervention	Dosage	Duration	Rationale
27/10/2022–03/11/2022	*Ama Pachana- Amritottaram Kashayam*	15 mL + 45mL warm water	Twice daily before food	*Deepana Pachana*
	*Vaishwanara Churnam*	1tsp -0-1tsp		
17/11/2022–	*Snehapana -*	1st day - 4th day –	4 days	
20/11/2022	*Indukanta Ghritam*	25 mL with warm water		
22/11/2022	*Mrdu Virechana Gandharvahastadi Eranda Tailam*	*-*10 mL with milk	1 Day	*Mrdu* *Shodhana- Pitta Vatahara*
25/11/2022 – 25/12/2022	*Drakshadi Kashayam*	20 mL + 60 mL of warm water morning and Evening before food.	1 month	*Vata Pittahara*
01/12/2022 – 15/12/2022	*Gandharvahastadi Eranda Tailam*	1 tsp with milk	15 days	*Vata Pittahara*

#### 6.2.2 External therapies

Medications used for external therapies included *Kottamchukkadi Tailam*, *Anu Tailam, Elaneer Kuzhambu, Triphala Kashayam, Ksheera Saindhava Seka, Shatahwadi* eye drops, *Triphala-Yashti Kashayam, Mukkadi Purambada, Shatahwadi Ghrita* and *Rasna Jambeera Svarasa*.

Refer to Table 5 for a complete list of medications used for external therapies.

**Table 5:** List of medications used for external therapies.

External therapies			
Date	Intervention	Medications	Dosage
21/11/2022	*Sarvanga Abhyanga* *+ Bashpa Sveda*	*Kottamchukkadi Tailam (Vatahara)*	100mL
26/11/2022–30/11/2022	*Marsha Nasya*	*Anu Tailam (Sroto Shodhana)*	6, 8, 10, 10,12 drops/day
01/12/2022 and 03/12/2022	*Anjana Kshalana*	*Elaneer Kuzhambu & Triphala Kashayam*	2 mL/day 200 mL/day
05/12/2022–08/12/2022	*Seka*	*Ksheera Saindhava*	400 mL/day
	*Ashchyotana*	*Shatahwadi* eye drops	10 mL/day
	*Pratimarsha Nasya*	*Anutaila*	2 drops/day
12/01/2023–20/01/2023	*Seka* (2 days)	*Triphala-Yasti Kashayam*	200 mL/day
	*Vidalaka* (2 days)	*Mukkadi Purambada*	1 Vati/day
	*Tarpana* (7 days)	*Shatahwadi Ghrita*	25 mL/day
	*Talam* (7 days)	*Rasna Jambeera Svarasa*	Q. S

#### 6.2.3 Changes in the therapeutic intervention

The treatment approach was dynamically adjusted based on the patient's response throughout the course. Initially, oral medications were administered to prepare the body by addressing *Ama Pachana* (therapeutic digestion of accumulated metabolites) before proceeding with *Vicharana Snehapana* (a method of co-administration of unctuous substances in various forms). Following *Snehapana*, the patient received *Abhyanga* for a day and was followed by *Mrdu Virechana* to correct the digestive/metabolic dysregulation. Subsequently, specific eye treatments, including *Marsha Nasya, Anjana, Kshalana* (irrigation)*, Seka, Ashchyotana* (instillation of liquid medicaments drop by drop in the eye)*, Vidalaka* (application of medicated paste on the outer part of eye)*, Tarpana* (optimal soothing collyrium)*,* and *Talam* (herbal paste or medicated oil to the bregma/crown of the head)*,* were done.

## 7 Follow-up and outcomes

### 7.1 Clinician and patient-assessed outcomes

#### 7.1.1 Clinician-assessed outcomes

During the patient's follow-up consultation after three months of outpatient treatment at the Department of Ophthalmology of the previously visited hospital, notable improvements were observed in dry eye condition without significant improvement in cataract-related visual acuity. Schirmer's test results showed increased tear production, with readings of 5 mm for the right eye and 7 mm for the left eye, which was previously noted to be 2 mm in the right eye and 3 mm in the left eye. Additionally, tear break-up time (TBUT) demonstrated improvement, measuring 6 s in the right eye and 8 s in the left eye, with no evidence of punctate keratopathy on ocular surface examination.

#### 7.1.2 Patient-assessed outcomes

An immediate outcome that the patient could appreciate was a reduction in the irritation, foreign body sensation and burning sensation. The OSDI score after the Ayurvedic treatment reduced to 10 from 50, which comes within the normal reference range. The patient also reported clarity in vision post-cataract surgery.

### 7.2 Relevant follow-up investigations and diagnostic findings

**At the biomedical hospital (22nd March 2023):** The patient's visual acuity was evaluated, revealing corrected vision of 6/60 (blurred) in both the right eye and left eye, improving to 6/6 (blurred) with pinhole correction. The right eye had a prescription of +2.75 Ds, while the left eye had a prescription of +3.00 Ds. Near vision correction was set at +2.75 Ds N8 for both eyes. Non-contact tonometry readings indicated intraocular pressures of 15 mmHg for the right eye and 13 mmHg for the left eye.

Keratometry readings were 43.75/43.75 at 180/90 for the right eye and 44.25/45.00 at 85/175 with a cylindrical correction of −0.75*85 for the left eye. A slit lamp examination revealed grade 2 nuclear sclerosis with posterior subcapsular cataract in both eyes. Schirmer's test recorded 5 mm in the right eye and 7 mm in the left eye. Dilated fundus examination showed a Cup-to-Disc Ratio (CDR) of 0.4 for the right eye and 0.6 for the left eye, with the rest of the fundus within normal limits. Due to the improvement in the ocular surface integrity, the patient was scheduled for cataract surgery.

### 7.3 Ayurvedic intervention adherence and tolerability

Although the patient reported strict adherence to the treatment regimen, treatment tolerance was assessed throughout the intervention. The patient was interrogated several times to find out if she encountered any difficulty in tolerating the Ayurvedic treatment (both internal medications and external therapies). The patient reported experiencing no discomfort throughout the treatment and informed that she tolerated it well.

### 7.4 Adverse and unanticipated events

No adverse or unanticipated events were reported by the patient or identified by the treating physician throughout the course of the Ayurveda treatment.

## 8 Discussion

The TFOS Dry Eye Workshop II (DEWS II, 2017) (24) defines dry eye as a multifactorial disease of the ocular surface characterised by a loss of tear film homeostasis, in which tear film instability, hyperosmolarity, ocular surface inflammation and damage, and neurosensory abnormalities play central etiological roles. Building on this concept, the TFOS DEWS III (2025) report (25) redefines dry eye as a multifactorial, symptomatic disease resulting from symptomatic homeostatic failure of the tear film and/or ocular surface. The updated framework emphasises driver-based aetiologies, including eyelid abnormalities, lacrimal dysfunction, neural impairment, and disturbances in the ocular surface microenvironment, recognising that alterations in cellular metabolism, immune regulation, and nerve function collectively contribute to disease pathophysiology.

To improve the consistency of dye eye diagnosis, TFOS DEWS III (2025) recommends a standardised two-level diagnostic algorithm. Level 1 focuses on identifying dry eye through symptom assessment and evaluation of tear film and ocular surface function. In the present case, this framework was adopted to provide a comprehensive assessment of disease severity and its underlying drivers. Symptom screening was performed using the OSDI, which evaluates the functional impact of dry eye symptoms, their frequency, and environmental triggers. Tear film instability, a hallmark feature of dry eye irrespective of its aetiology, was assessed using the TBUT test. Ocular surface staining was performed to detect epithelial damage and devitalised tissue, providing objective evidence of ocular surface compromise. Schirmer I testing was used to quantify basal and reflex aqueous tear production and to identify aqueous tear deficiency. Together, these validated assessments enabled a comprehensive evaluation of symptoms, tear film homeostasis, ocular surface integrity, and lacrimal gland function in accordance with the TFOS DEWS III (2025) diagnostic recommendations (26).

The principal clinical observation in this case was an improvement in both patient-reported symptoms and selected ocular-surface parameters following the multimodal Ayurvedic treatment. The OSDI score decreased from 50 to 10, while improvements were also observed in Schirmer's test I and TBUT. Improvement in ocular-surface integrity subsequently allowed the patient to proceed with cataract surgery. Nevertheless, because the intervention consisted of several systemic and local treatments administered sequentially, the contribution of any individual treatment component cannot be determined. In the present patient, markedly reduced Schirmer's values, shortened TBUT, punctate keratopathy and the absence of substantial meibomian gland abnormality were consistent with a predominantly aqueous-deficient phenotype. This clinical pattern is also consistent with published guidance on the diagnosis of ADDE (21).

ADDE results from inadequate aqueous tear production by the lacrimal functional unit and may occur in association with Sjögren's syndrome or through non-Sjögren causes such as ageing, systemic disease, medication use, lacrimal gland dysfunction, or environmental factors (21). Older age, female sex and diabetes were relevant predisposing factors in this patient. Previous studies have also shown that patients with non-Sjögren sicca may experience a symptom burden comparable to that of Sjögren-associated disease, even when the clinical signs differ in severity (5, 6). This emphasises the importance of evaluating patient-reported outcomes together with objective ocular-surface findings.

A notable patient-reported outcome in this case was the reduction in OSDI score from 50 to 10, corresponding to a 40-point absolute reduction and a shift from severe symptoms to the normal range. The reported minimal clinically important difference (MCID) for the OSDI is approximately 7.0–9.9 points across dry eye severity categories and 7.3–13.4 points among patients with severe symptoms at baseline (27). Thus, the improvement observed in this patient substantially exceeded the upper estimate of the MCID for severe dry eye. For context, the multicentre randomised DREAM trial reported mean OSDI reductions of 13.9 points in the omega-3 group and 12.5 points in the placebo group over 12 months (28), whereas a study of platelet-rich plasma eye drops reported a mean reduction of 8.6 points (29). Although the magnitude observed in the present case was numerically greater, these findings cannot be compared directly because of differences in study populations, interventions, follow-up periods, and study designs. Furthermore, as this is a single case involving multiple sequential interventions, the improvement should be interpreted as a marked and clinically meaningful patient-reported response temporally associated with the overall treatment protocol, including systemic and local ocular surface treatment, rather than evidence of efficacy or causality attributable to any individual component.

With regard to mechanistic studies, exploring the gut and eye connection, particularly in the context of *Ayurveda*, remains scarce. Until now, there is one case report and four clinical trials published on dry eye cases in Ayurveda but these papers have not evaluated the gut and ocular microbiome changes (30–35). This case report highlights the possibility of managing non-Sjögren's aqueous deficiency dry eye that is inadequately responsive to conventional biomedical interventions through an Ayurvedic treatment protocol focused on both systemic and ocular management, thereby improving ocular health. Experimental and observational studies suggest that intestinal dysbiosis may influence ocular-surface inflammation through systemic immune and metabolic pathways (18, 36–38) This provides biological plausibility for investigating a gut–eye relationship in dry eye. However, in this case, the gut microbiota composition, circulating inflammatory markers and ocular-surface inflammatory biomarkers were not evaluated. Consequently, this case cannot establish that the systemic treatment modified the gut microbiota or that such modification mediated the ocular improvement. The proposed gut–eye relationship should therefore remain a hypothesis for future investigation. The clinical presentation of lacrimal gland dysfunction-related non-Sjögren's aqueous deficiency dry eye matches the textual description of *Vata-Pittaja Shukshakshipaka*. The sequential treatment protocol comprised an initial systemic phase directed towards digestive and metabolic correction, followed by local ocular procedures and maintenance therapy. The classical rationale for the individual interventions and their sequence has been summarised in the intervention tables. These Ayurvedic constructs explain the clinical reasoning used to select the treatment; however, they should not be regarded as direct equivalents of lacrimal gland dysfunction, ocular-surface inflammation or other biomedical mechanisms. A clinically relevant observation from the sequential protocol-wise treatment administration was that ocular parameters remained largely unchanged during the initial gut-focused phase of treatment. Following the initiation of targeted ocular therapy in the subsequent phase, improvement was noted in both patient-reported symptoms and clinician-assessed findings. This temporal pattern, though, suggests an important contribution from the local treatment phase; however, it does not demonstrate that the systemic intervention restored the gut microbiota or facilitated the subsequent ocular response but only points to a possibility of an underlying mechanism working in the background. Loss of tear-film homeostasis, tear hyperosmolarity and ocular-surface inflammation are well-established mechanisms in dry eye disease according to TFOS DEWS II. By contrast, the gut–eye axis remains an emerging area of investigation.

### 8.1 Strengths

The biomedical diagnosis was established by a biomedical ophthalmologist. This case documents the potential role of Ayurvedic treatment in addressing non-Sjögren's ADDE, which was inadequately responsive to standard treatment in conventional medicine, and it is, to our knowledge, among the early documented case reports being treated, with an approach focusing on both the systemic and the local ocular intervention managing non-Sjögren's ADDE, emphasising the relevance of addressing gut imbalance along with ocular treatments rather than only treating the eye.

### 8.2 Limitations

(a) This is a single case report. A case series followed by well-controlled clinical trials is warranted to extrapolate the results to the larger population.
(b) As this manuscript was prepared retrospectively from routinely documented clinical records rather than prospectively for research purposes, only assessments performed as part of routine clinical practice, viz., the Schirmer's test I, TBUT, OSDI meibography, and fluorescein staining, which are ophthalmic test used to detect corneal epithelial damage or defects were used to confirm the diagnosis. Consequently, certain additional standardised diagnostic evaluations commonly recommended in clinical trials and research on dry eye such as the Oxford staining score, fluorescein staining grade, lissamine green staining, tear meniscus height, tear osmolarity and inflammatory biomarkers such as MMP-9 were not performed.
(c) As this case was retrospectively compiled from routine clinical records, slit-lamp photographs, baseline and follow-up fluorescein-staining images, tear meniscus images, and meibography images were unavailable. Furthermore, details of the fluorescein-staining protocol at the referring examination were not available, and this is a limitation. Therefore, differences in staining cannot be conclusively attributed to treatment response, examiner variability, or actual resolution of punctate keratopathy, and definitive conclusions regarding its resolution cannot be drawn.
(d) As the meibography before treatment was normal, there were practical difficulties in advising a post-treatment meibography, and this is a limitation in the documentation of this case report.
(e) Lens Opacities Classification System III (LOCS) grading was not performed during the original examination and limits the pre- and post-intervention comparison.
(f) The synergistic mechanism underlying the multimodal Ayurvedic interventions cannot be established from this single case, as gut and ocular microbiomes, microbial metabolites, and systemic or tear inflammatory biomarkers were not assessed; therefore, the proposed gut–eye interaction and any microbiome-mediated therapeutic effect remain hypothesis-generating and require validation through future mechanistic studies.

### 8.3 Future research directions

Future studies should test the hypothesis underlying this case report, based on the Ayurvedic principle of *Roga Marga* (pathways involved in disease manifestation and progression), wherein treatment targets both systemic and local factors implicated in dry eye.

There are preclinical studies and observational human studies that suggest possible associations between intestinal microbial composition, microbial metabolites and ocular-surface immune regulation, and from the observations of this particular case. However, it is not yet established that modification of the gut microbiota improves non-Sjögren's ADDE. Thus, studies in this direction will help in establishing these findings. Hence, future controlled studies should evaluate this sequential treatment approach using standardised OSDI assessment, tear osmolarity, ocular-surface staining, Schirmer's test I, TBUT and tear inflammatory biomarkers. If the gut–eye hypothesis is specifically investigated, paired gut and ocular microbiome assessments should be conducted before and after treatment.

### 8.4 The primary takeaway lessons of this case report

This case reports clinical improvement in severe, non-responsive dry eye following *Mrdu Shodhana* and subsequent *Kriyakalpa* in a cataract surgery candidate. Within the Ayurvedic framework, the observations may be viewed as consistent with favourable changes in *Agni* and *Bhrmhana Karma*; however, these interpretations remain hypothesis-generating and do not establish causality. The findings should therefore be understood as a single-case clinical observation rather than evidence of treatment efficacy.

## 9 Patient's perspective

“I have experienced considerable relief from my dry eye condition, particularly in terms of the discomfort characterised by foreign body sensations and a burning feeling in my eyes. I have ceased using teardrops. During a recent check-up with my former ophthalmologist, I underwent an examination and was deemed suitable for cataract surgery. I am profoundly thankful to the entire healthcare team for their dedicated and earnest efforts in helping me attain this milestone.”

This patient's perspective was recorded on 22/02/2023 after the completion of Ayurveda treatment.

## 10 Informed consent

Informed consent was obtained from the patient before initiating *Ayurvedic* treatment. Informed consent was separately obtained for publishing the details of her medical treatment.

## Data Availability

The datasets presented in this article are not readily available because they are clinical data of a patient treated by the author. The data supporting the conclusions of this article will be made available by the authors without undue reservation when required. Requests to access the datasets should be directed to Sushma Naranappa Salethoor, sushma@ay.amrita.edu.
